# Inadequate response to antiplatelet therapy in patients with peripheral artery disease: a prospective cohort study

**DOI:** 10.1186/s12959-022-00445-4

**Published:** 2023-01-10

**Authors:** B. M. M. Kremers, J. H. C. Daemen, H. ten Cate, H. M. H. Spronk, B. M. E. Mees, A. J. ten Cate-Hoek

**Affiliations:** 1 Department of Biochemistry, Laboratory for Clinical Thrombosis and Hemostasis, Maastricht University, Maastricht, The Netherlands; 2 Department of Vascular Surgery, Maastricht University Medical Center, Maastricht, The Netherlands; 3 Thrombosis Expertise Center, Maastricht University Medical Center, Maastricht, The Netherlands; 4 Department of Internal Medicine, Maastricht University Medical Center, Maastricht, The Netherlands; 5 Center for Thrombosis and Hemostasis, Gutenberg University Medical Center, Mainz, Germany

**Keywords:** Atherosclerosis, Atherothrombosis, Peripheral artery disease, Cardiovascular risk, Antiplatelet therapy, High on-treatment platelet reactivity

## Abstract

**Background:**

Patients with peripheral artery disease (PAD) are treated with preventive strategies to improve the cardiovascular risk. The incidence of cardiovascular events and mortality however remains high in PAD populations. We therefore aimed to better characterize PAD patients suffering from cardiovascular events and mortality in order to tailor preventive treatment.

**Methods:**

Between 2018 and 2020, 246 PAD outpatients (17 newly diagnosed, 229 with known PAD) were prospectively enrolled in this observational cohort study. Patient data and blood samples were collected after inclusion, and the primary composite endpoint (myocardial infarction, elective coronary revascularization, ischemic stroke, acute limb ischemia, mortality) was evaluated after one year. Secondary outcomes included platelet reactivity, measured using the VerifyNow assay, and medication adherence, assessed using the Morisky Medication Adherence Scale-8 (MMAS-8). Logistic regression models were used to identify associations between characteristics and the occurrence of events.

**Results:**

The cohort comprised 207 patients with claudication and 39 with chronic limb threatening ischemia. Twenty-six (10.6%) patients suffered from an event during follow-up. Prior myocardial infarction (OR 3.3 [1.4–7.7]), prior ischemic stroke (OR 4.5 [1.8–10.9]), higher levels of creatinine (OR 5.2 [2.2–12.6]), lower levels of high-density lipoprotein (OR 4.2 [1.5–10.6]) and lower haemoglobin levels (OR 3.1 [1.3–7.1]) were associated with events. Patients with events had more often high on-treatment platelet reactivity (HTPR) on aspirin (OR 5.9 [1.4–25.1]) or clopidogrel (OR 4.3 [1–19.3]). High adherence to medication was associated with the occurrence of events (OR 4.1 [1–18]).

**Conclusions:**

Patients suffering from cardiovascular events and mortality were characterized by prior cardiovascular events as compared to patients who did not experience any events. Antiplatelet therapy was not optimally protective despite high medication adherence, and HTPR was independently associated with the occurrence of events. More research is needed on alternative treatment strategies such as dual antiplatelet therapy or combinations with anticoagulant drugs.

**Trial registration:**

The Medical Ethics Committee (METC) of the MUMC+ approved the study (NL63235.068.17) and the study was registered in the Netherlands Trial Register (NTR7250).

## Background

Peripheral artery disease (PAD) is a vascular disease characterized by atherosclerosis-driven narrowing of peripheral arteries. The prevalence of PAD worldwide in individuals aged twenty-five years and older was estimated at 236 million in 2015 [1]. Despite its high prevalence, PAD remains underdiagnosed as many patients are asymptomatic and thus not aware of the disease [2]. However, both asymptomatic and symptomatic PAD patients are at risk of atherothrombotic events such as myocardial infarction and ischemic stroke with incidences of 15% over a period of three years [3, 4]. Within the symptomatic patient population, intermittent claudication, a mild manifestation of PAD, can be distinguished from the more severe chronic limb threatening ischemia. Intermittent claudication is classified as Fontaine II with typical symptoms of muscle pain during walking. Chronic limb threatening ischemia is classified as Fontaine III with rest pain and Fontaine IV with ischemic ulcer formation [5, 6]. PAD patients with chronic limb threatening ischemia are at a higher risk of adverse cardiovascular events with high mortality rates as compared to patients with intermittent claudication [4]. Current preventive strategies for cardiovascular events and mortality in PAD patients are based on risk management in which lipid-lowering drugs, antihypertensive drugs and antiplatelet drugs are the main treatment modalities. Statins are most widely used to improve the lipid profile targeted at a low-density lipoprotein (LDL) value of 1.8 mmol/L for PAD patients of 70 years or younger and a value of 2.5 mmol/L for PAD patients above 70 years [7]. By effectively lowering LDL levels, the incidence of cardiovascular events can be reduced significantly [8]. Addition of antihypertensive drugs to overcome hypertension as well as the use of antiplatelet drugs to effectively inhibit platelet activation reduces the incidence of cardiovascular events even further. Aspirin and clopidogrel are the antiplatelet drugs most often used as first-line treatment depending on national guidelines [5]. Although the CAPRIE-study demonstrated that clopidogrel was more effective than aspirin in reducing the combined risk of ischemic stroke, myocardial infarction, and cardiovascular death, this is no preferential treatment strategy [9]. Despite the established efficacy of antiplatelet regimes with regard to the reduction of cardiovascular events, high on-treatment platelet reactivity (HTPR) for both aspirin and clopidogrel may still occur and interfere with atheroprotective effects. HTPR is referred to as the failure of the antiplatelet agent to inhibit the target of its action [10, 11]. Aspirin HTPR prevalence is estimated at 17–26% in PAD populations [12–14] while clopidogrel HTPR appears to be more common with a prevalence up to 54% [12, 14–16]. The incidence of cardiovascular events in PAD populations remains high despite current treatment strategies [3]. Therefore, the aim of this observational cohort study was to better characterize PAD patients at risk of cardiovascular events and mortality in order to find targets for improved management.

## Methods

### Study design

Between May 2018 and May 2020, patients visiting the outpatient clinic of the department of Vascular Surgery of the Maastricht University Medical Center (MUMC+) were screened for PAD. Patients were eligible to participate in the study when the PAD was objectively diagnosed with an ankle-brachial index (ABI) of 0.9 or below. Fontaine II (intermittent claudication) and Fontaine III (chronic limb threatening ischemia) patients were selected and patients with Fontaine IV were excluded because of expected increased inflammatory parameters associated with ulcer formation. Further exclusion criteria were active malignancy, chronic inflammatory disease, coagulation disorders, pregnancy, age below 18, and the use of anticoagulant therapy. All eligible patients that were willing to participate were included after written informed consent was obtained. The Medical Ethics Committee (METC) of the MUMC+ approved the study (NL63235.068.17) and the study was registered in the Netherlands Trial Register (NTR7250; https://www.trialregister.nl/trial/7045).

### Blood collection and sample storage

Venous blood was drawn from the patients immediately after informed consent was signed. Blood drawing took place in a resting state and blood was collected by antecubital venipuncture with 21-gauge needles and 3.2% (w/v) citrated Vacutainer glass tubes, EDTA Vacutainer glass tubes and VACUETTE 9NC Coagulation 3.2% (w/v) Sodium Nitrate glass tubes. After blood drawing, the EDTA tubes and the citrate tubes were directly processed using the standard platelet-poor plasma centrifugation protocol used at our laboratory (4000 x g for 5 minutes followed by 11,000 x g for 10 minutes). Thereafter samples were, within two hours after blood drawing, frozen and stored at − 80° Celsius for further analysis. The VACUETTE 9NC tubes were immediately used to perform the VerifyNow assays for aspirin and clopidogrel.

### Data collection and measurements

Age, sex and date of PAD diagnosis of each patient were registered upon inclusion. The medical history of each patient including prior cardiovascular events such as myocardial infarction, ischemic stroke and PAD revascularization was collected from patient records. Each patient provided an updated medication list from which the use of lipid-lowering drugs, antihypertensive drugs and antiplatelet drugs were collected. The intensity of lipid-lowering strategies was categorized as high, medium and low intensity according to the ACC/AHA guideline [17]. Current smoking status, diabetes mellitus type 2 (DM2) and body mass index (BMI) were recorded. Patients were classified based on their symptoms upon inclusion using the Fontaine classification, and were then grouped as having intermittent claudication (Fontaine II) or chronic limb threatening ischemia (Fontaine III). The ABI at the time of diagnosis was measured and grouped by ratio as greater than 1.3 (incompressible), between 0.91 and 1.3, between 0.7 and 0.9, between 0.4 and 0.69 and below 0.4.

A complete blood cell count was performed at baseline and included levels of haemoglobin, haematocrit, thrombocytes and leukocytes with respective subpopulations. Platelet reactivity was assessed using the VerifyNow Aspi assay for Aspirin and VerifyNow P2Y12 assay for Clopidogrel (Accumetrics, San Diego, CA, USA). Blood collected in the VACUETTE 9NC tube was used in the optical detection system using a specific cartridge. The cut-off value for aspirin and clopidogrel HTPR was based on the most recent consensus document on the definition of on-treatment platelet reactivity, and was set at Aspirin Reaction Units (ARU) > 550 for aspirin [18] and P2Y12 Reaction Units (PRU) > 208 for clopidogrel [11]. Laboratory results that were collected from recent blood drawing included kidney function (creatinine, estimated glomerular filtration rate-Chronic Kidney Disease Epidemiology Collaboration (eGFR (CKD-EPI))), lipid profile (cholesterol, high-density lipoprotein (HDL), low-density lipoprotein (LDL), triglycerides) and haemoglobin A1c levels (HbA1c). The KDIGO guideline was used to classify the kidney function, and the stage of chronic kidney disease in each patient when appropriate [19].

Medication adherence was assessed by the licensed Morisky Medication Adherence Scale-8 (MMAS-8), which was developed by Morisky et al. The MMAS-8 is a validated assessment tool verified by numerous studies, consisting of eight questions to assess medication adherence [20–22]. Patients that were completely adherent scored a maximum score of 8, whereas the lowest possible adherence was scored 0. Each point decrease marked lower adherence to the medical treatment. According to MMAS-8 user guidelines the adherence was categorized as high (8 points), medium (7 or 6 points) and low (5 points or below).

### Outcome

The primary outcome consisted of a composite endpoint comprising myocardial infarction, ischemic stroke, acute limb ischemia, elective percutaneous intervention (PCI) or coronary artery bypass grafting (CABG) and all-cause mortality during the one-year follow-up. The outcome was assessed at 3, 6 and 12 months and was verified by telephone calls to the patient combined with hospital records. Patients who reached the composite endpoint were grouped as the “PAD event group” while patients who did not reach the composite endpoint were grouped as the “PAD no event group”. The secondary outcomes were platelet reactivity, HTPR and medication adherence.

### Statistical analysis

Baseline characteristics were collected for all patients and presented for patients with and without events during follow-up. Differences between both groups were analyzed using the chi-square test for dichotomous and categorical variables. For continuous variables, differences were analyzed using the parametric two-samples t-test or the non-parametric Mann-Whitney U test, as appropriate. Youden’s index was used, when appropriate, to determine optimal cut-off values for continuous variables. Univariable logistic regression models were used to test the associations of characteristics with the occurrence of events, reported as odds ratios with respective 95% confidence intervals (OR [95% CI]). Characteristics with an association with the occurrence of events (*p* < 0.05) in the univariable analysis were then used in multivariable models with backward stepwise logistic regression analysis for the occurrence of events, reported as odds ratios with respective 95% confidence intervals. Statistical significance was reached when *p* < 0.05. All analyses were performed using SPSS (IBM SPSS Statistics for Macintosh, Version 27.0. Armonk, NY: IBM Corp). All figures were created using GraphPad Prism (GraphPad Prism version 9 for Mac OS X, GraphPad Software, San Diego, California USA, www.graphpad.com).

## Results

### Patient characteristics

The cohort comprised 246 patients and baseline characteristics of the entire cohort as well as the distribution of patients with and without events are shown in Table 1. All patients had been diagnosed with PAD at a median of 33 (8–101) months prior to inclusion, while in 17 (6.9%) patients the diagnosis was established at the time of inclusion. Upon inclusion, most patients had intermittent claudication (207 (84.1%)) and 39 (15.9%) had chronic limb threatening ischemia. The cohort consisted of 141 (57.3%) male patients and the mean age was 68.7 ± 9.2 years. Most patients (109 (44.3%)) had an ABI between 0.7 and 0.9, while 99 (40.2%) and 21 (8.5%) patients had an ABI between 0.4 and 0.69 or below 0.4, respectively. The remaining 17 (6.9%) patients had incompressible arteries. Patient history revealed that 151 (61.4%) patients had previously undergone a peripheral revascularization procedure. Moreover, 72 (29.3%) patients had a prior myocardial infarction and 37 (15%) had a prior ischemic stroke. DM2 was present in 67 (27.2%) patients and the mean BMI was 26.4 ± 4.41 kg/m^2^. Of all patients, 229 (93.1%) had a history of smoking and 94 (38.2%) were current smokers with a median of 29 (15–40) pack years upon inclusion. A normal kidney function (G1) was observed in 41 (16.7%) patients, a mildly decreased kidney function (G2) in 138 (56.1%) patients, a mildly to moderately decreased kidney function (G3a) in 41 (16.7%) patients, a moderately to severe decreased kidney function (G3b) in 19 (7.7%) patients, a severely decreased kidney function (G4) in 6 (2.3%) patients and kidney failure (G5) in 1 (0.4%) patient.

**Table 1:** Baseline characteristics

	Total cohort (***n*** = 246)	Event group (***n*** = 26)	No event group (***n*** = 220)	***P***-value
	Mean ± SD / Median (IQR) / *n* (%)	Mean ± SD / Median (IQR) / *n* (%)	Mean ± SD / Median (IQR) / *n* (%)	
Age (years)	68.7 ± 9.2	71.5 ± 7.9	68.3 ± 9.3	0.093
Male gender	141 (57.3)	17 (65.4)	124 (56.4)	0.379
Newly diagnosed PAD patient upon inclusion	17 (6.9)	2 (7.7)	15 (6.8)	0.868
Chronic PAD patient upon inclusion	229 (93.1)	24 (92.3)	205 (93.2)	0.868
Time between diagnosis and inclusion (months)	33 (8–101)	81 (19–121)	26 (7–89)	0.028*
Intermittent claudication	207 (84.1)	22 (84.6)	185 (84.1)	0.945
Chronic limb ischemia	39 (15.9)	4 (15.4)	35 (15.9)	0.945
History of myocardial infarction	72 (29.3)	14 (53.8)	58 (26.8)	0.004*
History of stroke	37 (15)	10 (38.5)	27 (12.3)	0.001*
Current smoking	94 (38.2)	11 (42.3)	83 (37.7)	0.649
Pack years	29 (15–40)	33 (18–50)	29 (15–40)	0.175
BMI (kg/m^2^)	26.4 ± 4.41	27.4 ± 5.9	26.3 ± 4.2	0.390
DM2	67 (27.2)	10 (38.5)	57 (25.9)	0.174
HbA1c (mmol/mol)	43 (37–51)	43 (37–53)	43 (37–50)	0.752
Creatinine (μmol/L)	85 (72–104)	101 (77–145)	83 (71–101)	0.006*
eGFR (ml/min/1.73 m^2^)	71 (58–83)	59 (42–80)	72 (59–84)	0.022*
Haemoglobin (mmol/L)	8.66 ± 0.97	8.3 ± 1.1	8.7 ± 0.95	0.026*
Haematocrit (L/L)	0.42 ± 0.04	0.4 ± 0.04	0.42 ± 0.04	0.027*
Thrombocytes (× 10^3^ /mm^3^)	272 ± 87	277 ± 82	271 ± 88	0.714
Leukocytes (× 10^9^/L)	7.89 ± 2.2	8.44 ± 3.1	7.83 ± 2	0.342
Neutrophils (%)	62.2 ± 8.2	64 ± 7.5	62 ± 8.2	0.228
Lymphocytes (%)	25.8 ± 6.8	24.1 ± 6.5	26 ± 6.9	0.168
Eosinophils (%)	2 (1–3)	2 (2–3)	2 (1–3)	0.254
Basophils (%)	1 (1–1)	1 (1–1)	1 (1–1)	0.544
Monocytes (%)	8.83 ± 2.4	8.8 ± 3.2	8.8 ± 2.3	0.901
NLR	2.4 (1.9–3.1)	2.5 (2–3.4)	2.4 (1.9–3.1)	0.288
ABI at diagnosis				0.156
> 1.30 (incompressible)	17 (6.9)	0 (0)	17 (7.7)	
0.91–1.30	0 (0)	0 (0)	0 (0)	
0.70–0.90	109 (44.3)	9 (34.6)	100 (45.5)	
0.40–0.69	99 (40.2)	16 (61.5)	83 (37.7)	
< 0.40	21 (8.5)	1 (3.8)	20 (9.1)	

Baseline characteristics for the whole cohort and distribution between patients with and without cardiovascular events and mortality during follow-up. Significance was reached when *P* < 0.05 (*), significant values are in bold. *PAD* peripheral artery disease, *BMI* body mass index, *DM2* diabetes mellitus type 2, *HbA1c* haemoglobin A1c, *eGFR* estimated glomerular filtration rate, *NLR* neutrophil lymphocyte ratio, *ABI* ankle-brachial index, *SD* standard deviation, *IQR* interquartile range

### Composite endpoint

**Fig. 1:**
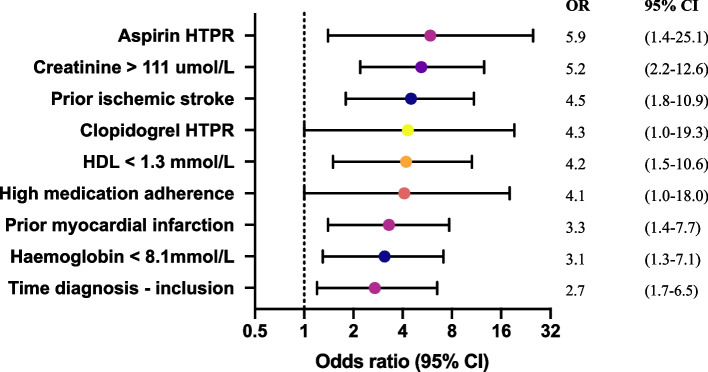
Univariable logistic regression analysis of characteristics associated with the occurrence of cardiovascular events and mortality, with corresponding odds ratios and 95% confidence intervals. HTPR = high on-treatment platelet reactivity, HDL = high-density lipoprotein, SD = standard deviation, IQR = interquartile range

All patients were followed for one year in which 26 (10.6%) patients reached the composite endpoint. Ten (38.5%) myocardial infarctions, four (15.4%) elective coronary revascularizations, five (19.2%) ischemic strokes and seven (26.9%) deaths were recorded. No differences were observed between patients with and without events regarding smoking status, DM2 and BMI. Both prior myocardial infarction (OR 3.3 [1.4–7.7]) and prior ischemic stroke (OR 4.5 [1.8–10.9]) were associated with the occurrence of events (Fig. 1). Also, decreased kidney function (plasma creatinine level > 111 μmol/L, OR 5.2 [2.2–12.6]) and plasma haemoglobin levels < 8.1 mmol/L (OR 3.1 [1.3–7.1]) were associated with the occurrence of events. Leukocyte and thrombocyte count were not associated*.*

### Evaluation of medication strategies

**Fig. 2:**
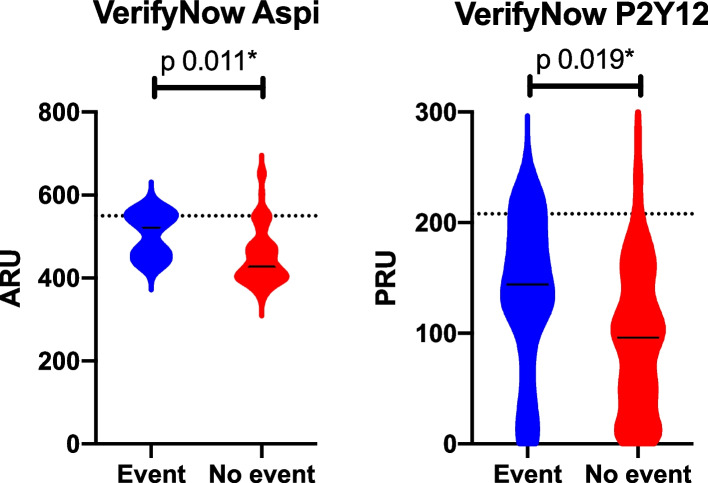
Platelet reactivity measured by the use of the VerifyNow assay in Aspirin Reactions Units (ARU) for aspirin users and P2Y12 Reaction Units (PRU) for clopidogrel users. Dotted lines represent HTPR which is an ARU > 550 for aspirin and a PRU > 208 for clopidogrel

**Fig. 3:**
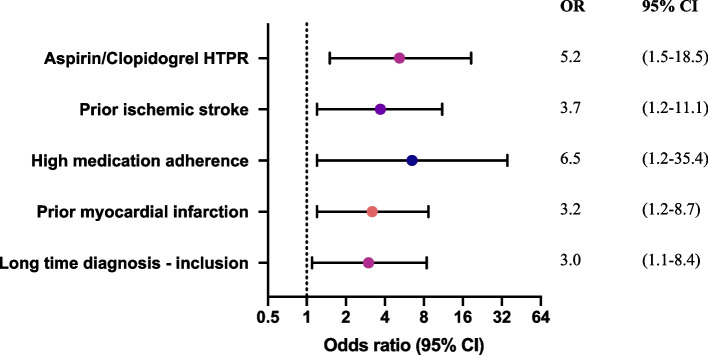
Multivariable logistic regression of characteristics associated with the occurrence of cardiovascular events and mortality, with corresponding odds ratios and 95% confidence intervals. HTPR = high on-treatment platelet reactivity, HDL = high-density lipoprotein, SD = standard deviation, IQR = interquartile range

All patients were treated according to current guidelines [5] which included the use of antihypertensive drugs, lipid-lowering drugs and antiplatelet drugs. The prescription of antihypertensive drugs (73.1% vs 72.7%, *p* = 0.834), lipid-lowering drugs (88.5% vs 90%, *p* = 0.396) and antiplatelet drugs (100% vs 100%, *p* = 1.000) did not differ between patients with and without events. Lipid-lowering strategies were prescribed in different intensities. A total of 71 (32.6%) patients were on high intensity lipid-lowering therapy without differences between patients with and without events (31.8% vs 32.7%, *p* = 0.937). Moderate and low intensity therapy had been applied in 138 (63.3%) and 9 (4.1%) patients, but also in these groups no differences were observed between patients with and without events (63.6% vs 63.3%, *p* = 0.973 and 4.5% vs 4.1%, *p* = 0.917 respectively). The effectiveness of the lipid-lowering therapies was assessed using the cholesterol profile, showing similar mean LDL levels of 2.44 ± 1.07 mmol/L between patients with events and those without (2.31 ± 1.14 mmol/L vs 2.45 ± 1.06 mmol/L, *p* = 0.542). In 58.5% of patients above 70 years old the LDL target level of 2.5 mmol/L was reached (2.49 ± 1.18 mmol/L), while the target level of 1.8 mmol/L was not reached in 95 (72%) patients 70 years or younger (2.39 ± 0.97 mmol/L). HDL levels were significantly lower in patients who experienced an event during follow-up (OR 4.2 [1.5–10.6]). The use of antiplatelet agents was evenly distributed in the cohort with 130 (52.8%) patients on aspirin, 127 (51.6%) on clopidogrel. Additionally, 11 (4.5%) patients were on dual antiplatelet therapy. During the conduct of this study, there was a transitioning of aspirin to clopidogrel as first choice antiplatelet agent in the hospital where patients were recruited. Therefore, some patients were using aspirin upon inclusion, while others were using clopidogrel. The median ARU on aspirin was 435 (402–482) and 12 (8.5%) patients had HTPR. The ARU in patients with events during follow-up was significantly higher compared to those without events (521 (452–554) vs 428 (401–478), *p* = 0.011) and HTPR was associated with the occurrence of events (OR 5.9 [1.4–25.1]). PRU in patients on clopidogrel were 100 (46–155) for the whole cohort and significantly higher in patients with events (144 (102–190) vs 96 (43–144), *p* = 0.019) (Fig. 2). HTPR on clopidogrel was observed in 8 (5.8%) patients and was associated with events (OR 4.3 [1–19.3])*.* In the multivariable analysis the adjusted OR for antiplatelet therapy was 5.2 [1.5–18.5] (Fig. 3).

High medication adherence was observed in 188 (76.4%) patients and was positively associated with the occurrence of events (OR 4.1 [1–18]). Medium adherence was observed in 46 (18.7%) patients and low adherence in 12 (4.9%) patients, both were not associated with the occurrence of events.

## Discussion

In this prospective observational cohort study, we characterized PAD patients with enhanced risk for cardiovascular events and mortality with the aim to find targets for improved management. Most patients in our cohort had prevalent PAD with a chronic state of atherosclerosis with years of plaque build-up and involvement of multiple vascular beds with associated decrease in renal function in conjunction with lower haemoglobin levels. Patients with such polyvascular disease are at increased risk for cardiovascular events with worsening prognosis when more vascular beds are affected [23]. In almost half of the patients in the cohort this polyvascular diseased state was present.

All patients in our cohort were treated with lipid-lowering agents, antiplatelet therapy and antihypertensive drugs. Interestingly, the medication prescribed did not appear to be sufficiently protective for PAD patients experiencing cardiovascular events and mortality, despite adequate adherence to the medication. Suboptimal target LDL levels were observed. The intensity of the lipid-lowering strategies prescribed was medium to high according to the ACC/AHA guidelines [7]. The LDL target level of 2.5 mmol/L was indeed reached in more than half of all patients older than 70 years. On the other hand, average LDL levels in patients of 70 years or younger were similar to the older patient group, while in these patients LDL levels of 1.8 mmol/L or lower are recommended. Especially in these younger patients the lipid-lowering regimen should ideally be intensified, which will likely result in a reduced incidence of cardiovascular events as the association between increased LDL levels and cardiovascular risk is well established [24]. Potentially, LDL target values of 1.8 and 2.5 mmol/L could even be lowered further as a recent study showed that lower concentrations of LDL may even better prevent cardiovascular events [25]. Also lower HDL levels were seen in patients that suffered from an event during follow-up, which indirectly supports the known atheroprotective effects of HDL including counteracting inflammation [26] and oxidative stress [27]. Several studies have found an association between lower HDL levels and cardiovascular risk in patients with coronary artery disease [28, 29] and low concentrations of HDL as one of the strongest lipoprotein risk factors for PAD [30, 31].

The VerifyNow assay was used to measure platelet reactivity while on aspirin or clopidogrel (or both). The residual platelet reactivity in patients on aspirin or on clopidogrel was significantly higher in patients experiencing events, indicating that platelets are less efficiently inhibited. Lack of medication adherence could have caused residual platelet reactivity. However, this did not seem to be the case as the results of the adherence score revealed that highly adherent patients were in the majority in the event group, which could be the result of increased awareness in this patient group as these patients more often experienced prior myocardial infarctions and ischemic strokes. Therefore, assuming that the adherence assessment is reliable, the current antithrombotic regime appears to be insufficient for adequate cardiovascular protection in these high-risk patients. Published studies show conflicting results regarding the association of HTPR with cardiovascular outcome. Two studies investigating clopidogrel HTPR found a significant association with cardiovascular events while two other studies did not [12, 14–16]. These studies used the same cut-off values for HTPR and follow-up duration was also similar. The contradicting results may however be explained by the lack of power. One study found a non-significant trend between clopidogrel HTPR and cardiovascular events [12], while the other study found a non-significantly increased hazard ratio in patients with HTPR [14]. In all four studies the prevalence of clopidogrel HTPR was higher as compared to the HTPR prevalence in our study, which could be explained by differences in medication adherence. We were not able to confirm this as other studies did not report on adherence. The strength of the risk association of HTPR that we found for both aspirin and clopidogrel suggests that optimization of antiplatelet therapy is an important management target for improvement. For aspirin, there is no known mechanism for biochemical resistance, but high platelet turnover could be a reason for residual platelet hyperreactivity [32]. In patients taking clopidogrel the HTPR could be explained by genetic polymorphisms in platelet receptor P2Y12 [33, 34] or polymorphisms of the CYP2C9 and CYP2C19 genes [35, 36]. Recent studies in patients with coronary artery disease investigated pharmacogenomics based on CYP2C19 gene variations to optimize therapy [37, 38]. Moreover, a meta-analysis concluded that the use of ticagrelor or prasugrel appeared more effective than clopidogrel in reducing the cardiovascular risk in patients with CYP2C19 gene variants [39]. Similar studies have yet to be performed in patients with PAD. The association between P2Y12 polymorphisms and the risk for cerebrovascular events in PAD patients has been established in the past [40]. Indeed, recent studies suggest that a twice-daily dosing of aspirin could improve its pharmacological efficacy. In patients with essential thrombocythemia a once-daily dose of aspirin as antithrombotic regime appeared inadequate in reducing platelet activation, while a dosing interval of 12 hours increased the antiplatelet response to aspirin [41, 42]. For clopidogrel, studies with increased dosing to compensate for the low inhibitory efficacy have been performed in the past [43], assuming “resistance” to be in part explained by too low concentrations of active clopidogrel at the platelet surface [44, 45]. However, apart from the use of loading doses in patients undergoing percutaneous coronary interventions, such regimens were never introduced in clinical practice in patients with PAD [46]. Dual antiplatelet therapy (DAPT) has been studied in the large CHARISMA trial [47]. Except for exceptionally thrombogenic conditions DAPT has not been introduced for long-term treatment of patients with PAD because of increased bleeding risk as compared to single antiplatelet therapy. Guidelines only recommend DAPT for a short period of time following percutaneous interventions and stenting in PAD. Several studies demonstrated that fibrinogen [48, 49] and d-dimer [50] levels were increased in high-risk PAD patients indicating an underlying hypercoagulable state. Anticoagulant treatment may counteract this prothrombotic state in PAD patients which is characterized by increased clot formation [51]. The COMPASS-trial has shown that dual pathway inhibition with aspirin and a low dose rivaroxaban reduced the incidence of cardiovascular events in high-risk PAD patients [52], suggesting that a reasonably low level of anticoagulation on top of antiplatelet therapy provides additional benefit. In spite of its demonstrated cost-effectiveness in at least a subset of PAD patients [53], the use of dual pathway inhibition in practice is still hindered by low uptake due to concerns about the number of pills per day in combination with an increased risk of major bleeding, even though fatal or critical organ bleeding events remained limited [52].

### Limitations

The use of the VerifyNow assay to identify HTPR may be perceived as a possible limitation of this study. Several studies have however shown that this assay correlates well with the “gold standard” of light transmission aggregometry for both aspirin [54] and clopidogrel [55]. The recorded rates of HTPR within our study population were lower than rates reported by most other studies [12, 14–16], this can however be explained by the overall high medication adherence rate that we recorded. The positive association that was found between high medication adherence and higher risk for cardiovascular events in the multivariable analysis may be confounded as this risk is likely to be primarily attributed to the higher rate of comorbidities and prior cardiovascular events leading to the increased motivation to be adherent to medication in these patients. Finally, due to sample size limitations, there is a lack of precision surrounding the estimates which demonstrates that there is still uncertainty about the actual effect size and that further information is needed.

## Conclusion

In our single-center cohort of PAD patients, current treatment strategies appeared to be insufficient for the reduction of cardiovascular risk. Lipid-lowering strategies should be intensified to further reduce LDL levels and improve the lipid profile. Antiplatelet agents were found to be inadequate despite high medication adherence, as platelet reactivity was insufficiently decreased in patients experiencing cardiovascular events. More research is needed on alternative treatment strategies such as dual antiplatelet therapy or combinations with anticoagulant drugs.

## Data Availability

Data will be available upon reasonable request by B. Kremers.

## Abbreviations

ABI: Ankle-brachial index
ARU: Aspirin reaction units
BMI: Body mass index
CABG: Coronary artery bypass grafting
DM2: Diabetes mellitus type 2
eGFR (CKD-EPI): Estimated glomerular filtration rate-Chronic Kidney Disease Epidemiology Collaboration
HbA1c: Haemoglobin A1c
HDL: High-density lipoprotein
HTPR: High on-treatment platelet reactivity
LDL: Low-density protein
METC: Medical Ethics Committee
MMAS-8: Morisky Medication Adherence Scale-8
MUMC: Maastricht University Medical Center
OR: Odds ratio
P2Y12: P2Y12 reaction units
PAD: Peripheral artery disease
PCI: Percutaneous intervention
